# Gender bias in shared decision‐making among cancer care guidelines: A systematic review

**DOI:** 10.1111/hex.13753

**Published:** 2023-04-05

**Authors:** Mario Rivera‐Izquierdo, Marta Maes‐Carballo, José J. Jiménez‐Moleón, Virginia Martínez‐Ruiz, Jan Blaakær, Rocío Olmedo‐Requena, Khalid S. Khan, Jan S. Jørgensen

**Affiliations:** ^1^ Departamento de Medicina Preventiva y Salud Pública Universidad de Granada Granada Spain; ^2^ Service of Preventive Medicine Hospital Universitario San Cecilio Granada Spain; ^3^ Instituto de Investigación Biosanitaria de Granada (ibs.Granada) Granada Spain; ^4^ Academic Department of General Surgery Complexo Hospitalario de Ourense Ourense Spain; ^5^ Academic Department of General Surgery Hospital Público de Verín Verín Spain; ^6^ Centro de Investigación Biomédica en Red de Epidemiología y Salud Pública (CIBERESP) Madrid España; ^7^ Research Unit of Gynaecology and Obstetrics, Department of Gynaecology and Obstetrics, Institute of Clinical Research, Odense University Hospital University of Southern Denmark Odense Denmark

**Keywords:** cancer diagnosis, cancer treatment, clinical guidelines, consensus statement, sex differences, shared decision‐making

## Abstract

**Background:**

In cancer care, the promotion and implementation of shared decision‐making in clinical practice guidelines (CPG) and consensus statements may have potential differences by gender.

**Objective:**

To systematically analyse recommendations concerning shared decision‐making in CPGs and consensus statements for the most frequent cancers exclusively among males (prostate) and females (endometrial).

**Search Strategy:**

We prospectively registered the protocol at PROSPERO (ID: RD42021241127). MEDLINE, EMBASE, Web of Science, Scopus and online sources (8 guideline databases and 65 professional society websites) were searched independently by two reviewers, without language restrictions.

**Inclusion Criteria:**

CPGs and consensus statements about the diagnosis or treatment of prostate and endometrial cancers were included from January 2015 to August 2021.

**Data Extraction and Synthesis:**

Quality assessment deployed a previously developed 31‐item tool and differences between the two cancers analysed.

**Main Results:**

A total of 176 documents met inclusion criteria, 97 for prostate cancer (84 CPGs and 13 consensus statements) and 79 for endometrial cancer (67 CPGs and 12 consensus statements). Shared decision‐making was recommended more often in prostate cancer guidelines compared to endometrial cancer (46/97 vs. 13/79, 47.4% vs. 16.5%; *p* < .001). Compared to prostate cancer guidelines (mean 2.14 items, standard deviation 3.45), compliance with the shared‐decision‐making 31‐item tool was lower for endometrial cancer guidelines (mean 0.48 items, standard deviation 1.29) (*p* < .001). Regarding advice on the implementation of shared decision‐making, it was only reported in 3 (3.8%) endometrial cancer guidelines and in 16 (16.5%) prostate cancer guidelines (*p* < .001).

**Discussion and Conclusions:**

We observed a significant gender bias as shared decision‐making was systematically more often recommended in the prostate compared to endometrial cancer guidelines. These findings should encourage new CPGs and consensus statements to consider shared decision‐making for improving cancer care regardless of the gender affected.

**Patient or Public Contribution:**

The findings may inform future recommendations for professional associations and governments to update and develop high‐quality clinical guidelines to consider patients' preferences and shared decision‐making in cancer care.

## 1 INTRODUCTION

The selection of the best diagnostic approach or treatment in cancer care must be personalized^1^, ^2^ given the vast quantity of strategies, screening techniques and therapeutical practices currently available.^3^ These decisions require a high level of patient participation.^4^ It has been purported that gender bias exists with the preferences of men being given greater priority than those of affected women.^5^

The participation of patients concerning the best diagnostic or treatment approach for their own disease through shared decision‐making (SDM) is currently considered essential in achieving sustainable, high‐quality cancer care.^4^, ^6^, ^7^, ^8^ This is important because different diagnostic or treatment options with similar potential may lead to different results depending on the patient's preferences and values.^4^, ^9^ SDM has been shown to increase patient satisfaction,^4^ cost‐effectiveness^4^ and reduce negligence claims.^10^ Therefore, in many developed countries, SDM is legally compulsory,^10^, ^11^, ^12^ and professional medical associations widely recommend it.^13^, ^14^, ^15^ The systematic implementation of SDM in cancer care faces several obstacles,^16^, ^17^, ^18^ and it is still poor.^19^, ^20^ Despite various proposed strategies to promote SDM,^9^, ^21^ clinical practice guidelines (CPGs) and consensus statements generally fail to recommend it, as recently suggested for breast cancer.^22^, ^23^ It is important to address the possible existence of a gender bias in SDM recommendations. This could be hypothesized for guidelines concerning cancers that exclusively affect biological males versus those that exclusively affect biological females. Particularly, major implications can result from treating prostate cancer, such as disruptions to urinary, bowel or sexual function. Due to the significant tradeoffs with prostate cancer screening and treatment, SDM has been strongly encouraged.^2^, ^14^ In fact, according to the US Preventive Services Task Force, screening of prostate cancer using the prostate‐specific antigen (PSA) presents grade C of evidence for men aged 55–69 years (meaning that the decision should be individualized), and grade D for men older than 69 years, which has led to a reduction in the screening.^24^

Similarly, the treatment of endometrial cancer can result in significant consequences, such as loss of fertility for premenopausal females, urinary or faecal incontinence or early menopause, among others. Decisions regarding hormonal treatment after oophorectomy for perimenopausal females remain uncertain. The significant counterparts of the screening and surgical or hormonal treatment of this pathology, also make SDM highly recommendable for its diagnosis and treatment. Again, the US Preventive Services Task Force highlights that there is no standard or routine screening test for endometrial cancer and all of them have risks and side effects, including periodic pelvic examination.^25^

We systematically reviewed the characteristics of CPGs and consensus statements concerning SDM in the diagnosis and treatment of the most frequent cancer exclusively affecting males, that is, prostate cancer, and the most frequent cancer exclusively affecting females, that is, endometrial cancer.

## 2 PATIENTS AND METHODS

The systematic review was conducted following prospective protocol registration (Prospero ID: CRD42021241127) and was reported according to the Preferred Reporting Items for Systematic Reviews and Meta‐Analyses^26^ (Supporting Information: Appendix 1). For comparison, we selected the most frequent exclusively male cancer (prostate cancer)^27^ and the most frequent exclusively female cancer (endometrial cancer, also known as uterine cancer, carcinoma of the uterine corpus or adenocarcinoma of the endometrium).^27^

### 2.1 Search strategy and data source

We conducted a systematic search covering from January 2015 to August 2021, combining MeSH terms ‘shared decision‐making’, ‘clinical practice guidelines’, ‘guidelines’, ‘consensus’, ‘prostate cancer’, ‘prostate cancer diagnosis’, ‘prostate cancer treatment’, ‘endometrial cancer’, ‘endometrial cancer diagnosis’ and ‘endometrial cancer treatment’, and including word variants in TRIP database and MEDLINE, without language restrictions. We started the search in 2015 given that the recommended period for updating CPGs is every 5 years. Subsequently, we extended the search to other databases, such as EMBASE, Web of Science, Scopus, Cochrane Database of Systematic Reviews and the ACP Journal Club. Eight guideline databases were searched, including National Institute for Health and Care Excellence (NICE), National Comprehensive Cancer Network (NCCN), Scottish Intercollegiate Guidelines Network, Fisterra, Canadian CPG or CMA Infobase, National Health and Medical Research Council, Health Services Technology Assessment Texts and Guidelines International Network. Finally, 99 relevant professional society websites were visited to complete the search (Supporting Information: Appendix 2), and references from systematic reviews and other studies on this topic were analysed.

### 2.2 Study selection and data extraction

We covered CPGs and consensus statements on diagnosis and therapeutic management of prostate or endometrial cancer, developed by professional societies, organizations or government agencies. Guidelines on the management of cancer complications (e.g., castration‐resistant prostate cancer, or Lynch syndrome for endometrial cancer) were also included. Obsolete documents updated in more recent years from the same organization, documents for education or information purposes (only if they specified so or if it was only an infographic) and documents designed only for patients (only if they specified so) were omitted. The titles and abstracts identified in the search were assessed by two independent reviewers (M. R.‐I. and V. M.‐R.) as well as a full‐text assessment of the selected studies to confirm eligibility. Potential disagreements or inconsistencies were resolved by consensus with a third reviewer (M. M.‐C.). Duplicate documents were removed. The management of the information (selected documents) for the review was facilitated using EndNote® version 20 (Clarivate Analytics).

### 2.3 Quality assessment

The 31‐item tool^23^ for quality assessment of CPGs and consensus statements on SDM was used, originally based on items identified from the AGREE II^28^ and RIGHT^29^ tools, and SDM bibliography of interest (Supporting Information: Appendix 3). The consensus meeting following approval of the 31‐item checklist recommended that each individual item should be examined for compliance so that a greater number of items fulfilled means higher quality for SDM in the CPGs or consensus statements assessed. The selected studies were assessed independently by two reviewers (M. R.‐I. and V. M.‐R.), and disagreements were resolved by the consensus of a third reviewer (M. M.‐C.). The quality assessment was divided into 13 domains (Supporting Information: Appendix 3). No formal score or cut point for defining quality was considered, as recommended by the authors of the tool.^23^

### 2.4 Statistical analyses

First, a descriptive analysis of quality assessment items concerning SDM was conducted separately for prostate and endometrial cancer. Second, differences between both groups were analysed using *T* tests, and *χ* ^2^ tests for quantitative and qualitative variables, respectively. When *χ* ^2^ conditions for applications were not met, Fisher exact tests were applied.

## 3 RESULTS

### 3.1 Study selection

**Figure 1:**
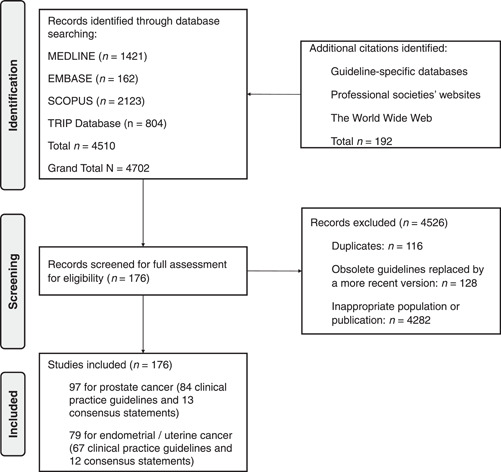
Flowchart of the article selection process.

Of the 4702 identified citations on the search, 176 met inclusion criteria, 97 for prostate cancer (84 CPGs and 13 consensus statements) and 79 for endometrial cancer (67 CPGs and 12 consensus statements) (Figure 1). Of the total, 84 (47.7%) were published in a journal^30^, ^31^, ^32^, ^33^, ^34^, ^35^, ^36^, ^37^, ^38^, ^39^, ^40^, ^41^, ^42^, ^43^, ^44^, ^45^, ^46^, ^47^, ^48^, ^49^, ^50^, ^51^, ^52^, ^53^, ^54^, ^55^, ^56^, ^57^, ^58^, ^59^, ^60^, ^61^, ^62^, ^63^, ^64^, ^65^, ^66^, ^67^, ^68^, ^69^, ^70^, ^71^, ^72^, ^73^, ^74^, ^75^, ^76^, ^77^, ^78^, ^79^, ^80^, ^81^, ^82^, ^83^, ^84^, ^85^, ^86^, ^87^, ^88^, ^89^, ^90^, ^91^, ^92^, ^93^, ^94^, ^95^, ^96^, ^97^, ^98^, ^99^, ^100^, ^101^, ^102^, ^103^, ^104^, ^105^, ^106^, ^107^, ^108^, ^109^, ^110^, ^111^, ^112^, ^113^ and 93 were published in other sources (Supporting Information: Appendix 4).

### 3.2 Characteristics of the studies

Table 1 shows the main characteristics of the selected documents, including the title, year and country. There was a total of 67 (38.1%) European documents, 65 (36.9%) North American documents, 18 (10.2%) Asian documents, 11 (6.3%) South American documents, 6 (3.4%) Oceanian documents and 5 (2.8%) African documents. The remaining seven (4.0%) documents were from international organizations that brought together countries from different continents. From the total selected documents, 90 (51.1%) were diagnostic guidelines and 140 (79.5%) were therapeutic guidelines (therefore, several documents included information on both diagnostic and therapeutic approaches).

**Table 1:** Clinical guidelines and consensus statements on diagnosis and treatment of prostate cancer (*n* = 97) and endometrial cancer (*n* = 79), 2015–2021.

Name of the clinical practice guideline	Entity	Country	Year
*Prostate cancer*			
PMB definition guideline: Prostate cancer	CMS	South Africa	2020
South African prostate cancer guidelines	SAUA	South Africa	2017
Update of Guidelines for Management of Prostate Cancer in West Africa 2019: Consensus Working Document	WA	West Africa	2019
NCCN Asia Consensus Statement prostate cancer	NCCN	Asia	2018
Chinese guidelines for diagnosis and treatment of prostate cancer 2018	NHC China	China	2018
Chinese Expert Consensus on the Diagnosis and Treatment of Castration‐Resistant Prostate Cancer (2019 Update)	CEC	China	2019
Consensus statements on the management of clinically localized prostate cancer from the Hong Kong Urological Association and the Hong Kong Society of Uro‐Oncology	HKUA‐HKSUO	China	2019
Expert Group Consensus Opinion on Prostate Cancer Diagnosis and Management in India	Consensus	India	2020
Evidenced‐based clinical practice guideline for prostate cancer (summary: Japanese Urological Association, 2016 edition)	JUA	Japan	2016
2020 Korean guidelines for the management of metastatic prostate cancer	KSMO	Korea	2020
Prostate cancer	MIMS	Malaysia	2021
Singapore Cancer Network (SCAN) Guidelines for the Management of Advanced Castrate‐Resistant Prostate Cancer	SCAN	Singapore	2015
Saudi Oncology Society and Saudi Urology Association combined clinical management guidelines for prostate cancer 2017	SOS‐SUA	Saudi Arabia	2017
EAU‐EANM‐ESTRO‐ESUR‐SIOG Guidelines on Prostate Cancer—2020 Update. Part 1: Screening, Diagnosis, and Local Treatment with Curative Intent	EAU‐EANM‐ESTRO‐ESUR‐SIOG	Europe	2020
EAU‐EANM‐ESTRO‐ESUR‐SIOG Guidelines on Prostate Cancer. Part II—2020 Update: Treatment of Relapsing and Metastatic Prostate Cancer	EAU‐EANM‐ESTRO‐ESUR‐SIOG	Europe	2020
Biochemical recurrence in prostate cancer: The EAU Prostate Cancer Guidelines Panel's recommendations	EAU‐EANM‐ESTRO‐ESUR‐SIOG	Europe	2020
ESMO Clinical Practice Guidelines for diagnosis, treatment and follow‐up of prostate cancer	ESMO	Europe	2020
Guidelines on Prostate Cancer	EAU‐ESTRO‐ESOR‐SIOG	Europe	2018
EAU‐ESTRO‐SIOG Guidelines on prostate cancer: screening, diagnosis and local treatment with curative intent	EAU‐ESTRO‐SIOG	Europe	2017
DUCG's National Guidelines for Diagnosis and Treatment of Prostate Cancer	DUCG	Denmark	2015
French ccAFU guidelines—update 2020‐2022: prostate cancer	CCAFU	France	2020
S3—Prostate cancer guideline	AWMF‐DKG‐DKH	Germany	2021
PSMA ligand PET/CT in the diagnosis of prostate carcinoma	AWMF	Germany	2019
National Prostate Cancer GP Referral Guideline	NCCP	Ireland	2018
Diagnosis, staging and treatment of patients with prostate cancer. National Clinical Guideline No. 8	NCCP	Ireland	2016
Prostate cancer, national guideline version 3.0	IKNL	Netherlands	2017
Appropriate use of pharmaceutical products for patients with castration‐refractory prostate cancer	Zorginstituut Nederland	Netherlands	2016
Prostate cancer	NVU	Netherlands	2016
SEOM clinical guidelines for the treatment of advanced prostate cancer (2020)	SEOM	Spain	2020
SEOM clinical guidelines for the treatment of metastatic prostate cancer (2017)	SEOM	Spain	2017
Enzalutamide for treating hormone‐sensitive metastatic prostate cancer (technology appraisal guidance TA712)	NICE	UK	2021
Darolutamide with androgen deprivation therapy for treating hormone‐relapsed non‐metastatic prostate cancer (technology appraisal guidance TA660)	NICE	UK	2020
Guidance for the assessment and management of prostate cancer treatment induced bone loss. A consensus position statement from an expert group	Expert group	UK	2020
Prostate cancer: diagnosis and management (NICE guideline NG131)	NICE	UK	2019
Enzalutamide for hormone‐relapsed non‐metastatic prostate cancer (Technology appraisal guidance TA580)	NICE	UK	2019
Padeliporfin for untreated localised prostate cancer (Technology appraisal guidance TA546)	NICE	UK	2018
Memokath‐051 stent for ureteric obstruction (Medical technologies guidance MTG35)	NICE	UK	2018
Prostate cancer screening with prostate‐specific antigen (PSA) test: a clinical practice guideline	MAGIC‐BMJ	UK	2018
Biodegradable spacer insertion to reduce rectal toxicity during radiotherapy for prostate cancer (Interventional procedures guidance IPG590)	NICE	UK	2017
Irreversible electroporation for treating prostate cancer	NICE	UK	2016
Interventional procedures guidance [IPG572]			
Radium‐223 dichloride for treating hormone‐relapsed prostate cancer with bone metastases (Technology appraisal guidance TA412)	NICE	UK	2016
Cabazitaxel for hormone‐relapsed metastatic prostate cancer treated with docetaxel (Technology appraisal guidance TA391)	NICE	UK	2016
Degarelix for treating advanced hormone‐dependent prostate cancer (Technology appraisal guidance TA404)	NICE	UK	2016
Abiraterone for castration‐resistant metastatic prostate cancer previously treated with a docetaxel‐containing regimen (Technology appraisal guidance TA259)	NICE	UK	2016
Abiraterone for treating metastatic hormone‐relapsed prostate cancer before chemotherapy is indicated (Technology appraisal guidance TA387)	NICE	UK	2016
Enzalutamide for treating metastatic hormone‐relapsed prostate cancer before chemotherapy is indicated (Technology appraisal guidance TA377)	NICE	UK	2016
Suspected cancer: recognition and referral (NICE guideline NG12)	NICE	UK	2015
Brachytherapy for Patients With Prostate Cancer: American Society of Clinical Oncology/Cancer Care Ontario Joint Guideline Update	ASCO/CCOJ	USA/Canada	2017
Canadian consensus forum of key controversial areas in the management of advanced prostate cancer	GURC	Canada	2021
Canadian Urological Association guideline on androgen deprivation therapy: Adverse events and management strategies	CUA	Canada	2021
Canadian Urological Association best practice report: Prostate‐specific membrane antigen positron emission tomography/computed tomography (PSMA PET/CT) and PET/magnetic resonance (MR) in prostate cancer	CUA	Canada	2021
2021 Canadian Urological Association (CUA)‐Canadian Uro‐Oncology Group (CUOG) guideline: Management of castration‐resistant prostate cancer (CRPC)	CUA	Canada	2021
Multiparametric Magnetic Resonance Imaging in the Diagnosis of Clinically Significant Prostate Cancer. Guideline 27‐2 version 2	CCO	Canada	2021
A Canadian framework for managing prostate cancer during the COVID‐19 pandemic: Recommendations from the Canadian Urologic Oncology Group and the Canadian Urological Association	CUA	Canada	2020
Canadian Urological Association‐Canadian Urologic Oncology Group guideline on metastatic castration‐naive and castration‐sensitive prostate cancer	CUA	Canada	2020
Current topics in radiotherapy for genitourinary cancers: Consensus statements of the Genitourinary Radiation Oncologists of Canada	GUROC	Canada	2020
Local prostate cancer. Clinical Practice Guideline GU‐012—Version 3	CCA	Canada	2020
Advanced/Metastatic prostate cancer. Clinical Practice Guideline GU‐010—Version 2	CCA	Canada	2020
Prostate Cancer Part 1: Diagnosis and Referral in Primary Care	BC	Canada	2020
Prostate Cancer Part 2: Follow‐up in Primary Care	BC	Canada	2020
Canadian consensus algorithm for erectile rehabilitation following prostate cancer treatment	CUA	Canada	2018
An Endorsement of the 2018 Guideline on Hypofractionated Radiation Therapy for Localized Prostate Cancer: An ASTRO, ASCO, and AUA Evidence‐Based Guideline	CCO	Canada	2018
Guideline for Optimization of Surgical and Pathological Quality Performance for Radical Prostatectomy in Prostate Cancer Management. Evidence‐Based Series 17‐3 Version 2	CCO	Canada	2017
Canadian Urological Association recommendations on prostate cancer screening and early diagnosis	CUA	Canada	2017
Cancer Care Ontario Position Statement on Prostate Cancer Screening using the Prostate Specific Antigen (PSA) Test	CCO	Canada	2017
Follow‐up Care for Survivors of Prostate Cancer—Clinical Management: a Program in Evidence‐Based Care Systematic Review and Clinical Practice Guideline	CCO	Canada	2017
Multiparametric magnetic resonance imaging for pre‐treatment local staging of prostate cancer: A Cancer Care Ontario clinical practice guideline	CCO	Canada	2016
Bone Health and Bone‐Targeted Therapies for Prostate Cancer. Guideline 3‐14 Version 2	CCO	Canada	2016
Prostate cancer, 2015.	CCA	Canada	2015
Society for Immunotherapy of Cancer (SITC) clinical practice guideline on immunotherapy for the treatment of urothelial cancer	SITC	USA	2021
Initial Management of Noncastrate Advanced, Recurrent, or Metastatic Prostate Cancer: ASCO Guideline Update	ASCO	USA	2021
Advanced prostate cancer: AUA‐ASTRO‐SUO guideline	AUA‐ASTRO‐SUO	USA	2020
Bone Health and Bone‐Targeted Therapies for Prostate Cancer: ASCO Endorsement of a Cancer Care Ontario Guideline	ASCO	USA	2020
Prostate cancer: NCCN Clinical Practice Guidelines in Oncology	NCCN	USA	2019
Prostate cancer early detection. NCCN Clinical Practice Guidelines in Oncology	NCCN	USA	2019
Incontinence after Prostate Treatment: AUA/SUFU Guideline (2019)	AUA‐SUFU	USA	2019
Adjuvant and Salvage Radiotherapy after Prostatectomy: ASTRO/AUA Guideline	ASTRO‐AUA	USA	2019
Prostate cancer prevention and early detection	ACS	USA	2019
Castration‐resistant prostate cancer	AUA	USA	2018
Screening for Prostate Cancer: US Preventive Services Task Force Recommendation Statement	USPSTF	USA	2018
Early detection of prostate cancer: AUA guideline	AUA	USA	2018
Clinically Localized Prostate Cancer: ASCO Clinical Practice Guideline Endorsement	ASCO	USA	2018
ASTRO/ASCO/AUA Guideline on Hypofractionation for Localized Prostate Cancer	ASTRO‐ASCO‐AUA	USA	2018
American Joint Committee on Cancer. Prostate	AJCC	USA	2017
Clinically Localized Prostate Cancer: AUA‐ASTRO‐SUO Guideline.	AUA‐ASTRO‐SUO	USA	2017
Second‐Line Hormonal Therapy for Men With Chemotherapy‐Naïve, Castration‐Resistant Prostate Cancer: ASCO Provisional Clinical Opinion	ASCO	USA	2017
Role of Genetic Testing for Inherited Prostate Cancer Risk: Philadelphia Prostate Cancer Consensus Conference 2017	PPCCC	USA	2017
NCCN Clinical Practice Guidelines in Oncology (NCCN Guidelines). Version 3.	NCCN	USA	2016
Radiotherapy for recurrent prostate cancer: 2018 Recommendations of the Australian and New Zealand Radiation Oncology Genito‐Urinary group	FROGG	Australia and New Zealand	2018
Clinical practice guidelines: PSA Testing and Early Management of Test‐Detected Prostate Cancer	PCFA	Australia and New Zealand	2016
AUGE Clinical Guidelines. Prostate cancer in patients over 15 years old	MSC	Chile	2015
Prostate cancer. Risk factors, early detection and PSA: screening, use and correct interpretation	AMUC	Costa Rica	2018
Prostate cancer diagnosis and treatment. Clinical practice guidelines	IMSS	Mexico	2018
Clinical practice guideline: prostate cancer	AUNA	Peru	2019
Clinical practice guideline for the screening, diagnosis and treatment of localized and locally advanced prostate cancer	IETSI	Peru	2021
Clinical Practice Guideline for the early detection, diagnosis, staging, treatment, rehabilitation and follow‐up of patients with prostate cancer.	INEN	Peru	2021
Management of patients with advanced prostate cancer: APCCC consensus conference	APCCC	International	2019
*Endometrial/uterine cancer*			
Cancer of the uterus	CANSA	South Africa	2021
PMB definition guideline: Endometrial cancer	CMS	South Africa	2019
Chinese expert consensus on fertility‐preserving treatment for young women with early stage well differentiated endometrial cancer	CRHA	China	2021
Consensus document for management of uterine cancer	ICMR	India	2019
Japan Society of Gynecologic Oncology 2018 guidelines for treatment of uterine body neoplasms.	JSGO	Japan	2018
Practice guidelines for management of uterine corpus cancer in Korea: a Korean Society of Gynecologic Oncology Consensus Statement	KSGO	Korea	2017
Singapore Cancer Network (SCAN) Guidelines for the Systemic Therapy of Endometrial (Uterine) Cancer	SCAN	Singapore	2015
Management of histologically confirmed endometrial cancer JE/003/21	SLCOG	Sri Lanka	2021
ESGO/ESTRO/ESP Guidelines for the management of patients with endometrial carcinoma.	ESGO‐ESTRO‐ESP	Europe	2021
Endometrial Cancer MRI staging: Updated Guidelines of the European Society of Urogenital Radiology	ESUR	Europe	2019
ESMO‐ESGO‐ESTRO Consensus Conference on Endometrial Cancer: diagnosis, treatment and follow‐up	ESMO‐ESGO‐ESTRO	Europe	2016
European Society of Gynecological Oncology Task Force for Fertility Preservation: Clinical Recommendations for Fertility‐Sparing Management in Young Endometrial Cancer Patients	ESGO	Europe	2015
Cancer patients follow‐up—Croatian Society of Medical Oncology Part I: breast cancer, uterine cancer, cervical cancer, ovarian cancer	CSMO	Croatia	2016
Surgical treatment of endometrial cancer	DGCG	Denmark	2021
Guidelines for the referral, diagnosis, treatment, and control of cancer of the uterine corpora.	DGCG	Denmark	2019
4th revision of the guideline			
In which cases should endometrial destruction be performed during an operative hysteroscopy? Clinical practice guidelines from the French College of Gynaecologists and Obstetricians (CNGOF)	CNGOF	France	2021
Nice‐Saint‐Paul de Vence 2020 recommendations for clinical practice: Management of metastatic and/or relapsing endometrial cancer	ARCAGY‐GINECO	France	2020
Recommendations for the surgical management of gynecological cancers during the COVID‐19 pandemic—FRANCOGYN group for the CNGOF	CNGOF	France	2020
Primary management of endometrial carcinoma. Joint recommendations of the French society of gynecologic oncology (SFOG) and of the French college of obstetricians and gynecologists (CNGOF)	SFOG‐CNGOF	France	2017
Cancer early detection policy (KFE‐RL)	GB	Germany	2020
Current recommendations for surveillance, risk reduction and therapy in Lynch syndrome patients	GCFIC	Germany	2019
Guideline on the Diagnosis, Treatment, and Follow‐up of Patients with Endometrial Cancer	GGP (AWMF‐DKG‐DKH)	Germany	2018
Interdisciplinary Diagnosis, Therapy and Follow‐up of Patients with Endometrial Cancer. Guideline (S3‐Level, AWMF Registry Nummer 032/034‐OL, April 2018)—Part 1 with Recommendations on the Epidemiology, Screening, Diagnosis and Hereditary Factors of Endometrial Cancer	AWMF	Germany	2018
Interdisciplinary Diagnosis, Therapy and Follow‐up of Patients with Endometrial Cancer. Guideline (S3‐Level, AWMF Registry Number 032/034‐OL, April 2018)—Part 2 with Recommendations on the Therapy and Follow‐up of Endometrial Cancer, Palliative Care, Psycho‐oncological/Psychosocial Care/Rehabilitation/Patient Information and Healthcare Facilities	AWMF	Germany	2018
Dutch National Guideline Endometrial Cancer Version 3.1 [Guideline]	RCGO‐IKNL	Netherlands	2018
Uterine cancer (endometrial cancer)	NGF	Norway	2021
Project for the National Program of Early Diagnosis of Endometrial Cancer Part I	PEDEC	Romania	2015
Project for the National Program of Early Diagnosis of Endometrial Cancer Part II	PEDEC	Romania	2015
SEOM clinical guidelines for endometrial cancer (2017)	SEOM	Spain	2017
Joint RCOG/BGCS Guidance for Care of Patients with Gynaecological Cancer during the COVID‐19 Pandemic	RCOG‐BGCS	UK	2021
Implementing Lynch syndrome testing and surveillance pathways	NHS	UK	2021
National optimal pathway to endometrial cancer: Point of suspicion to first definitive treatment in adults (aged 16 and over)	GCSG‐GIG‐NHS	UK	2020
Guidance for radiotherapy for gynaecological cancer and COVID‐19	RCR	UK	2020
Testing strategies for Lynch syndrome in people with endometrial cancer. Diagnostics guidance [DG42]	NICE	UK	2020
All Wales Guideline for the Management of Uterine Cancer	GCSG‐GIG‐NHS	UK	2019
Sentinel Consensus Document for Vulval, Endometrial and Cervical Cancer BGCS	BGCS	UK	2019
The Manchester International Consensus Group recommendations for the management of gynecological cancers in Lynch syndrome	MICG	UK	2019
Endometrial Cancer Clinical Quality Performance Indicators	SNTF‐NCQSG	UK	2018
BGCS Uterine Cancer Guidelines: Recommendations for Practice	BGCS	UK	2017
Standards and datasets for reporting cancers. Dataset for histological reporting of endometrial cancer	RCPATH	UK	2017
Management of uterine cancers	GOGG‐MCGCNG	UK	2016
Guideline for the Management of Endometrial Cancer Formerly the Guideline for Post Menopausal Bleeding and Endometrial Cancer	PBCN‐NHS	UK	2015
Systemic Therapy for Advanced or Recurrent Endometrial Cancer and Advanced or Recurrent Uterine Papillary Serous Carcinoma	CCO	Canada	2019
Princess Margaret Cancer Centre. Clinical Practice Guidelines. Gynecologic cancer: Endometrial	UHN PMCC	Canada	2019
Endometrium	BC Cancer Agency	Canada	2018
Screening for Lynch Syndrome by Immunohistochemistry BRAF Mutations Analysis and MLH1 Promoter Methylation Analysis for Patients in Ontario with Colorectal or Endometrial Cancers	CCO	Canada	2015
Endometrial cancer. Clinical practice guideline GYNE‐002 Version 5	AHS	Canada	2015
NRG Oncology/RTOG Consensus Guidelines for Delineation of Clinical Target Volume for Intensity Modulated Pelvic Radiation Therapy in Postoperative Treatment of Endometrial and Cervical Cancer: An Update	NRGO‐RTOG	USA	2021
ACR Appropriateness Criteria® Pretreatment Evaluation and Follow‐Up of Endometrial Cancer	ACR	USA	2020
Use of cannabinoids in cancer patients: A Society of Gynecologic Oncology (SGO) clinical practice statement	SGO	USA	2020
Endometrial intraepithelial neoplasia	ACOG	USA	2019
Gynecological Cancers Translational, Research Implementation, and Harmonization: Gynecologic Cancer InterGroup Consensus and Still Open Questions	GCI	USA	2019
The American Brachytherapy Society consensus statement for electronic brachytherapy	ABS	USA	2018
Uterine Neoplasms, Version 1.2018, NCCN Clinical Practice Guidelines in Oncology	NCCN	USA	2018
ASTRO Guideline on the Role of Postoperative Radiation Therapy for Endometrial Cancer	ASTRO	USA	2017
Opioid Use in Gynecologic Oncology; Balancing Efficacy, Accessibility and Safety: An SGO Clinical Practice Statement	SGO	USA	2017
An update on post‐treatment surveillance and diagnosis of recurrence in women with gynecologic malignancies: Society of Gynecologic Oncology (SGO) recommendations	SGO	USA	2017
Diagnosis and management of endometrial cancer	AFP	USA	2016
Adjuvant Management of Early Stage Endometrial Cancer	ACR	USA	2016
Postoperative Radiation Therapy for Endometrial Cancer: American Society of Clinical Oncology Clinical Practice Guideline Endorsement of the American Society for Radiation Oncology Evidence‐Based Guideline	ASCO‐ASRO	USA	2015
Practice Bulletin. Clinical management guidelines for obstetrician‐gynecologists: Endometrial cancer	ACOG	USA	2015
Consensus statement for brachytherapy for the treatment of medically inoperable endometrial cancer	ABS	USA	2015
Society of Gynecologic Oncology statement on risk assessment for inherited gynecologic cancer predispositions	SGO	USA	2015
SGO clinical practice statement: the role of sentinel lymph node mapping in endometrial cancer	SGO	USA	2015
Shared follow‐up care for women with low‐risk endometrial cancer: A guide for General Practitioners (GP Guide)	CA	Australia	2020
Shared follow‐up and survivorship care for women with low‐risk endometrial cancer: summary of evidence	CA	Australia	2020
Gynaecological cancer: A guide to clinical practice in NSW	NSWG‐ACI	Australia	2019
Clinical practice guidelines for the treatment and management of endometrial cancer	CA	Australia	2016
Brazilian Society of Surgical Oncology guidelines for surgical treatment of endometrial cancer in regions with limited resources	BSSO	Brazil	2020
Consensus Committee Federación Argentina de Sociedades de Ginecología y Obstetricia F.A.S.G.O. Consenso de Ginecología FASGO 2019 ‘Endometrial Cancer’	FASGO	Argentina	2019
Inter‐Societies National Consensus on Endometrial Cancer	CIIS‐ANM	Argentina	2016
Endometrial Cancer Management Guideline Protocol	SCGO	Chile	2018
Consensus of the Oncological Gynaecology Branch of the Chilean Society of Obstetrics and Gynaecology			
Proposed diagnostic, staging and surgical protocol for endometrial cancer	IOMPC	Venezuela	2018
Endometrial Carcinoma, Grossing and Processing Issues: Recommendations of the International Society of Gynecologic Pathologists	ECTF‐ISGyP	International	2019
International Society of Gynecological Pathologists (ISGyP) Endometrial Cancer Project: Guidelines From the Special Techniques and Ancillary Studies Group	ISGyP	International	2019
Endometrial Carcinoma Diagnosis: Use of FIGO Grading and Genomic Subcategories in Clinical Practice: Recommendations of the International Society of Gynecological Pathologists	ISGP	International	2019
Endometrial cancer histopathology reporting guide	ICCR	International	2017
Guidelines for pre‐ and intra‐operative care in gynecologic/oncology surgery: Enhanced Recovery After Surgery (ERAS®) Society recommendations—Part I	ERAS Society	International	2015
Guidelines for pre‐ and intraoperative care in gynecologic/oncology surgery: Enhanced Recovery After Surgery (ERAS®) Society recommendations—Part II	ERAS Society	International	2015

*Note*: The guidelines are presented divided by cancer, continent, country and year.

Abbreviations: NICE, National Institute for Health and Care Excellence; NCCN, National Comprehensive Cancer Network.

### 3.3 Factors associated with SDM

Only 59 (33.5%) guidelines included information on SDM. Table 2 shows the characteristics of the guidelines stratified by the presence of SDM. The studies published in 2018 and after were characterized by a higher frequency of SDM reporting than studies conducted before 2018 (*p* = .010). The country, the publication in a journal and the nature of the guideline (diagnostic or therapeutic) were not associated with the presence of SDM for the total sample. Regarding prostate cancer guidelines, diagnostic guidelines, mainly focused on screening using PSA, were characterized by a higher frequency of SDM than therapeutic guidelines (*p* = .057). Regarding endometrial cancer guidelines, European documents were distinguished by a higher frequency of addressing SDM than non‐European guidelines (*p* = .003).

**Table 2:** Characteristics of the clinical practice guidelines (CPGs) and consensus statements (CSs) stratified by the presence of shared decision‐making (SDM).

Characteristics	CPGs and CSs with SDM	CPGs and CSs without SDM	*p* Value*
Total sample (*n* = 176)	59 (33.5%)	117 (66.5%)	–
Year of publication			
Published in 2018 and after	45 (40.5%)	66 (59.5%)	.010
Published before 2018	14 (21.5%)	51 (78.5%)	
Type of document			
CPGs	52 (34.4%)	99 (65.6%)	.528
CSs	7 (28.0%)	18 (72.0%)	
Continent			
European guidelines	25 (37.3%)	42 (62.7%)	.404
North American guidelines	21 (32.3%)	44 (67.7%)	.794
South American guidelines	4 (36.4%)	7 (63.6%)	.837
Asian guidelines	5 (27.8%)	13 (72.2%)	.586
Oceanian guidelines	3 (50.0%)	3 (50.0%)	.665
African guidelines	2 (40.0%)	3 (60.0%)	.763
Publication in a journal			
Published in a journal	28 (33.7%)	55 (66.3%)	.955
Not published in a journal	31 (33.3%)	62 (66.7%)	
Focus of the guideline			
Diagnostic guidelines	35 (38.9%)	55 (61.1%)	.123
Therapeutic guidelines	45 (32.1%)	95 (67.9%)	.444
Prostate cancer (*n* = 97)	46 (47.4%)	51 (52.6%)	–
Year of publication			
Published after 2018	35 (56.5%)	27 (43.5%)	.018
Published before 2018	11 (31.4%)	24 (68.7%)	
Type of document			
CPGs	40 (47.6%)	44 (52.4%)	.922
CSs	6 (46.2%)	7 (52.8%)	
Continent			
European guidelines	15 (42.9%)	20 (57.1%)	.499
North American guidelines	20 (50.0%)	20 (50.0%)	.670
Asian guidelines	5 (41.7%)	7 (58.3%)	.670
Publication in a journal			
Published in a journal	25 (52.1%)	23 (47.9%)	.363
Not published in a journal	21 (42.9%)	28 (57.1%)	
Focus of the guideline			
Diagnostic guidelines	26 (57.8%)	19 (42.2%)	.057
Therapeutic guidelines	34 (43.6%)	44 (56.1%)	.1262
Endometrial cancer (*n* = 79)	13 (16.5%)	66 (83.5%)	–
Year of publication			
Published after 2018	10 (20.4%)	39 (79.6%)	.350
Published before 2018	3 (10.0%)	27 (90.0%)	
Type of the document			
CPGs	12 (17.9%)	55 (82.1%)	.679
CSs	1 (8.3%)	11 (91.7%)	
Continent			
European guidelines	10 (31.3%)	22 (68.8%)	.003
North American guidelines	1 (4.0%)	24 (96.0%)	.052
South American guidelines	0 (0.0%)	5 (100.0%)	.584
Asian guidelines	0 (0.0%)	6 (100.0%)	.582
Publication in a journal			
Published in a journal	6 (13.3%)	39 (86.7%)	.389
Not published in a journal	7 (20.6%)	27 (79.4%)	
Focus of the guideline			
Diagnostic guidelines	9 (20.0%)	36 (80.0%)	.328
Therapeutic guidelines	11 (17.7%)	51 (82.3%)	.723

* *p* Value of *χ* ^2^ test or Fisher exact test, when appropriate.

### 3.4 Factors associated with the type of cancer

**Figure 2:**
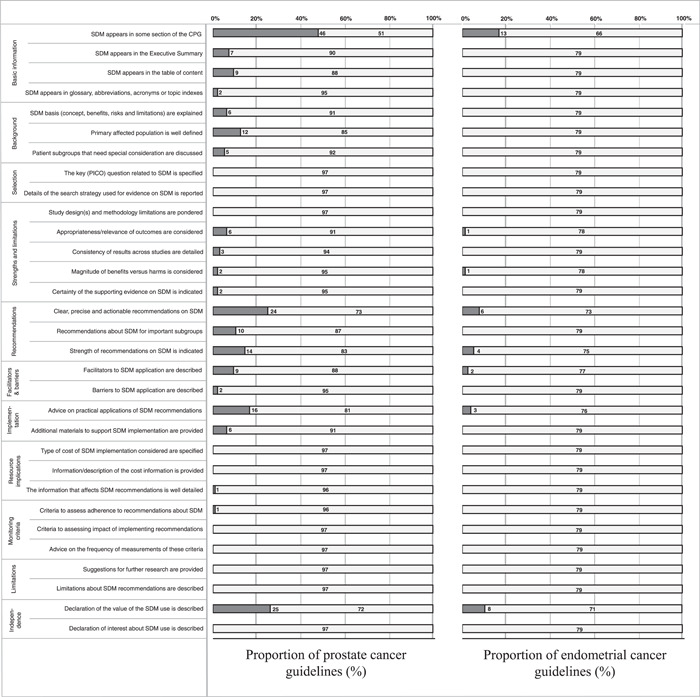
Compliance of the data extraction items of prostate and endometrial cancer guidelines.

A total of 46 (47.4%) prostate cancer guidelines addressed SDM, contrasting with 13 (16.5%) endometrial cancer guidelines (*p* < .001). Complete information on differences between prostate and endometrial cancer guidelines is shown in Table 3. When applying the 31‐item tool^23^ for assessing SDM compliance of the data extraction items (Figure 2), we showed important differences depending on the cancer type. Although compliance with the items was low for both types of cancer, most of them were much lower for endometrial cancer. No item presented a higher frequency of compliance with the endometrial cancer guidelines. Prostate cancer guidelines demonstrated a mean score of 2.14 points (standard deviation of 3.45); a median of 0 (interquartile range: 0–3); a range of 0–16 points. Endometrial cancer guidelines presented a mean score of 0.48 points (standard deviation 1.29); median of 0 (interquartile range 0–0), range of 0–5 points. Regarding the guidelines that reported SDM, the mean score for prostate cancer documents was 4.48 (standard deviation 3.85), and the mean score for endometrial cancer was 2.92 (standard deviation 1.76) (*p* = .043).

**Table 3:** Characteristics of the clinical practice guidelines (CPGs) and consensus statements (CSs) stratified by cancer (prostate cancer and endometrial cancer).

Characteristics	Prostate cancer CPGs and CSs (*n* = *97)*	Endometrial cancer CPGs and CSs (*n* = 79)	*p* Value*
*Groups*			
Presence of shared decision‐making	46 (47.4%)	13 (16.5%)	<.001
Number of shared decision‐making items: mean (standard deviation)^a^	2.14 (3.45)	0.48 (1.29)	<.001
Year of publication			
Published in 2018 or after	62 (63.9%)	49 (62.0%)	.796
Published before 2018	35 (36.1%)	30 (38.0%)	
Type of document			
CPGs	84 (86.6%)	67 (84.8%)	.735
CSs	13 (13.4%)	12 (15.2%)	
Continent			
European guidelines	35 (36.1%)	32 (40.5%)	.548
North American guidelines	40 (41.2%)	25 (31.6%)	.190
South American guidelines	6 (6.2%)	5 (6.3%)	.969
Asian guidelines	12 (12.4%)	6 (7.6%)	.298
Oceanian guidelines	2 (2.1%)	4 (5.1%)	.410
African guidelines	3 (3.2%)	2 (2.6%)	.828
Publication in a journal			
Published in a journal	48 (49.5%)	45 (57.0%)	.323
Not published in a journal	49 (50.5%)	34 (43.0%)	
Focus of the guideline			
Diagnostic guidelines^b^	45 (46.4%)	45 (57.0%)	.163
Therapeutic guidelines	78 (80.4%)	62 (78.5%)	.752

^a^ Items of shared decision‐making quality assessment in CPGs and CSs according to the 31‐item tool developed by Maes‐Carballo et al.^23^

^b^ Diagnostic and treatment guidelines account for more than 100% of the percentage as several documents were both diagnostic and treatment guidelines.

* *p* Value of *χ* ^2^ test or Fisher exact test, when appropriate. For the variable ‘number of shared decision‐making items’, *T* test was applied.

When analysing individual items, SDM did not appear in executive summaries, tables of content or glossaries in endometrial cancer clinical practice guidelines and consensus statements, while appeared in 7.2% (*p* = .017), 9.3% (*p* = .005) and 2.1% (*p* = .502) of prostate cancer documents. Similarly, SDM basis (concept, benefit, risks and limitations), primary affected population and patient subgroups that need special consideration were assessed by none of the endometrial cancer guidelines and addressed by 6.2% (*p* = .033), 12.4% (*p* = .001) and 5.2% (*p* = .065) of the prostate cancer guidelines, respectively. Regarding selection criteria, no document complied with any of the explored items. The strengths and limitations of SDM were poorly covered by both prostate and endometrial cancer guidelines (<3% for each item). A clear recommendation on SDM was one of the most considered items (24.7% of prostate cancer documents and 7.6% of endometrial cancer documents) (*p* = .003). Nevertheless, separated recommendations for important subgroups and an indication of the strength of the recommendation on SDM were scarcely detailed in endometrial cancer guidelines (0.0% and 5.1%) compared to prostate cancer guidelines (10.3%, *p* = .003% and 14.3%, *p* = .041, respectively). Facilitators to SDM applications, barriers, advice on practical application and additional material to support SDM implementation were described in 9.3%, 2.1%, 16.5% and 6.2% of prostate cancer documents, but only addressed in 2.5%, 0.0%, 3.8% and 0.0% of endometrial cancer documents. There was an absence of data on resource implications, monitoring or evaluating criteria for SDM, limitations and conflict of interest regarding SDM in all guidelines. Finally, a declaration of the value of the SDM use was described in 25.8% of prostate cancer and 10.1% (*p* = .008) of endometrial cancer documents.

## 4 DISCUSSION

Our thorough systematic review of clinical practice guidelines and consensus statements regarding prostate and endometrial cancer diagnosis and treatment found that recommendations concerning SDM were not universal for both types of cancer. Importantly, however, they were significantly weaker for endometrial versus prostate cancer, demonstrating a gender bias in SDM for cancer care. We found that recommendations on SDM were more frequent in recent guidelines, but important items regarding SDM reporting (especially those regarding advice on SDM implementation) were missing across the time horizon.

We chose two diseases (prostate cancer and endometrial cancer) for which SDM is especially recommended. The risks and benefits involved in treatment decisions are uncertain and should be individualized. As an example, according to the most recent prostate cancer guideline provided by the European Association of Urology,^41^ radical prostatectomies should not be denied on the grounds of age alone, but the stage of the disease, the frailty of the patient and the consensus between specialists and the patient should guide the final decision. This is also applicable to active surveillance, watchful waiting or radiotherapy, among other treatment options. Postoperative incontinence and erectile dysfunction are common problems following surgery, around 20% and 70%, respectively.^41^ Therefore, risks and benefits must be considered and discussed, and the management of complications should be equally approached. Similarly, surgery as a treatment for endometrial cancer can also lead to fertility loss, urinary incontinence or early menopause. According to the most recent European guideline,^80^ several options can be discussed, such as ovarian preservation, minimally invasive surgery or other treatments aimed to preserve fertility, according to the clinical situation (stage of the disease, comorbidities, etc.) and the desires of the patient. More information on treatment options, risks and benefits for these procedures is available from the clinical guidelines selected in this review. Nevertheless, SDM might not be perceived as a priority for policymakers as it is not added as reimbursable action. Similarly, organizations may not have SDM as a priority area for options that are equally old versus nascent. Therefore, there may be an underlying bias that exists outside the scope of this review, partially explaining the low frequency of SDM found in clinical guidelines. Similarly, potential differences in the state of recommendations on screening and treatment options depending on the type of cancer might also affect the interpretation of results. It is important to note that this work is focused on a potential gender bias regarding SDM in CPG of cancer affecting different biological sexes (as a proxy for potential differences based on social, cultural or psychological issues). Future specific studies should analyze and discuss whether the differences found in our study might reflect actual gender bias in cancer care.

A key strength of our study was a global perspective with a large number of clinical practice guidelines and consensus statements included. We did not restrict our search to specific languages or data source limitations. Nevertheless, it should be noted that gender bias is not equal across the world, which might influence the results of this review. We tried to approach this point by comparing the frequency of SDM in different continents, but no important differences were observed. One perceived limitation of our study is the subjective nature of the data extraction regarding SDM reporting of the selected documents. We tried to minimize this issue by using duplication data extraction with the arbitration. The quality assessment tool might be a further issue, as the items considered had been given the same relevance and weight, whereas future research should score them creating a threshold for rating quality.^23^ Prostate and endometrial cancers might not be representative of all exclusively male and exclusively female cancers. Therefore, as prostate cancer presents a blood test with a biomarker (PSA) that is not highly specific and endometrial cancer has not, a higher need for SDM in prostate cancer might be needed. Nevertheless, every patient needs to be part of the decision when choosing between treatment alternatives. In our study, when comparing treatment guidelines, that present numerous alternatives of similar efficacy for both cancers, the differences in favour of prostate cancer remain. We only included guidelines from 2015 to date, to avoid a selection bias as SDM is increasingly implemented in current guidelines and given that most of the guidelines before that date have been updated and replaced by new ones.

We found that half of prostate cancer clinical practice guidelines and consensus statements considered SDM, compared with only a sixth of endometrial cancer clinical practice guidelines and consensus statements. As the most frequent exclusively male and exclusively female cancers, these differences might represent the tip of the iceberg for the presence of a gender bias in patients' participation and self‐decision on their disease's diagnostic and treatment approaches. Our data regarding SDM sex differences are underpinned by other studies on breast cancer,^23^ reporting 40.5% of SDM in breast cancer documents, lower than prostate cancer data. Moreover, when analysing the 31 items regarding the quality of reporting and compliance with the data extraction, we observed that none of the items was significantly higher in breast cancer than in prostate cancer. Although breast cancer exists in males (and, therefore, these guidelines are not exclusive to females), most breast cancer patients are females. We found a surprisingly low frequency of SDM in endometrial cancer care, although being the most frequent cancer that exclusively affects females in the world^80^, ^100^ and has a wide variety of treatment options, especially depending on the female's fertility desires and stage of the disease.^80^, ^100^ We also showed a lower frequency of SDM in non‐European gynaecological guidelines, which suggests that further information and dissemination on SDM benefits should be especially strengthened in these contexts. We showed that SDM is increasingly being covered in guidelines in the most recent years. Most of the guidance methodological handbooks for updating clinical practice guidelines recommend that the time between updates should be 2 or 3 years, therefore older guidelines run the risk of being outdated.^114^ We only covered prostate and endometrial cancer guidelines for comparing a potential gender bias in SDM in cancer care, as the most frequent exclusively male and female cancers requiring SDM according to the recommendations, due to feasibility criteria. Sex disparities in this regard should be confirmed by studying other exclusively‐men cancers (e.g., testicular cancer) and other exclusively female cancers (e.g., cervical, or ovarian cancer). Potential differences in recommendations may reflect a bias in the statement of clinical evidence for men versus women (e.g., grade of recommendation of screening for both pathologies). Although elucidating that gap is not within the scope of this project, we recommend approaching this point in future research, not only for cancer care.

Our results suggest that SDM should be introduced in endometrial cancer guidelines, and also reinforced in prostate cancer guidelines. SDM must be present in future updated clinical practice guidelines and consensus statements of any cancer in which diagnostic or treatment options have similar potential, regardless of the gender affected. As SDM could positively influence the diagnosis and prognosis of cancer and the lack of studies on this topic, it will be necessary to adequately cover SDM in these documents, especially those published in a medical journal or widely accepted by a professional society. Patient preferences and desires must be taken into account and SDM should be considered in any cancer care guidance. The practical implications of our results are that endometrial and gynaecological cancer guidelines require a deep reflection on how to introduce SDM for improving patient care.

## 5 CONCLUSIONS

SDM was recommended in around a half and a fifth of prostate and endometrial cancer guidelines respectively. Several items concerning SDM study selection, resource implications, implementation, monitoring criteria and limitations, have not been reported to date in any prostate or endometrial cancer guideline. Compared to endometrial cancer, prostate cancer documents covered more recommendations on SDM, advice on practical applications of SDM and declaration of the value of SDM use. Thus, there is a gender bias that merits further investigation and correction to achieve equality in improving cancer care.

## AUTHOR CONTRIBUTIONS

Each author certifies that he/she has made a direct and substantial contribution to the conception and design of the study, the development of the search strategy, the establishment of the inclusion and exclusion criteria, data extraction, analysis and interpretation. Mario Rivera‐Izquierdo, Khalid S. Khan and Jan S. Jørgensen designed the work. Mario Rivera‐Izquierdo, Marta Maes‐Carballo and Virginia Martínez‐Ruiz collected and interpreted the data for the work and analysed the data. Mario Rivera‐Izquierdo wrote the first version of the draft. Mario Rivera‐Izquierdo, Marta Maes‐Carballo, José J. Jiménez‐Moleón, Virginia Martínez‐Ruiz, Jan Blaakær, Rocío Olmedo‐Requena, Khalid S. Khan and Jan S. Jørgensen revised the work critically for important intellectual content. Marta Maes‐Carballo, José J. Jiménez‐Moleón and Khalid S. Khan supervised the work. All authors approved the final version of the manuscript and agreed to be accountable for all aspects of the work in ensuring that questions related to the accuracy or integrity of any part of the work are appropriately investigated and resolved.

## CONFLICT OF INTEREST STATEMENT

The authors declare no conflict of interest.

## Supporting information

Supplementary information.

Supplementary information.

Supplementary information.

Supplementary information.

## Data Availability

The authors confirm that the data supporting the findings of this study are available within the article and its Supporting Information: Materials.
