# Supplementary material for: Gender bias in shared decision‐making among cancer care guidelines: A systematic review
Source: Health Expect. 2023 Apr 5;26(3):1019–38. doi: 10.1111/hex.13753 (PMC10154819; doi:10.1111/hex.13753)
Supplement: Supplementary file 2 — Supplementary information. [file HEX-26--s001.docx]

**Appendix 2**

**Data sources and search strategy**

*Sample search strategy for Medline*

We conducted a systematic search on 15 August 2021 in MEDLINE (via PubMed; from January 2015 to August 2021) using the following combination of free-text terms:

For prostate cancer:

#1 Practice guideline [pt]

#2 Practice guidelines as topic [mesh]

#3 Guideline [pt]

#4 guidelines as topic [mesh]

#5 consensus [mesh]

#6 OR #1-#5

#7 prostate neoplasms [mesh]

#8 prostate neoplasms [all]

#9 prostate cancer [mesh]

#10 prostate cancer [all]

#10 OR #7-9

#11 2015 [pdta] : 3000[pdta]

# #6 AND # 10 AND #11

Results: 881 articles

For endometrial cancer:

#1 Practice guideline [pt]

#2 Practice guidelines as topic [mesh]

#3 Guideline [pt]

#4 guidelines as topic [mesh]

#5 consensus [mesh]

#6 OR #1-#5

#7 endometrial neoplasms [mesh]

#8 endometrial neoplasms [all]

#9 endometrial cancer [mesh]

#10 endometrial cancer [all]

#10 OR #7-9

#11 2015 [pdta] : 3000[pdta]

# #6 AND # 10 AND #11

Results: 540 articles

*Online databases*

1. MEDLINE
2. EMBASE
3. Web of Science
4. Scopus
5. The Cochrane Database of Systematic Reviews
6. Cochrane Methodology Register
7. ACP Journal Club
8. Database of Abstracts of Reviews of Effects
9. Cochrane Central Register of Controlled Trials (CENTRAL)
10. The Health Technology Assessment

*Guideline-specific databases*

1. NHMRC, Australia
2. CMA Infobase, Canada
3. CPG, Canada
4. GIN, International
5. NZGG, New Zealand
6. NICE, UK
7. Trip Database, UK
8. SIGN, UK
9. Fisterra, Spain
10. HSTAT, USA
11. NCCN, USA
12. NGC, USA

*Professional societies*

1. FASGO, Argentina
2. ANM, Argentina
3. Australian Government, Australia
4. FROGG, Australia & New Zealand
5. PCFA, Australia & New Zealand
6. KCE, Belgium
7. BSSO, Brazil
8. Alberta Health Services, Canada
9. CancerCare Manitoba, Canada
10. CCO & Ontario Ministry of Health, Canada
11. CCOJ, Canada
12. GUROC, Canada
13. Ministerio de Salud de Chile, Chile
14. SCGO, Chile
15. Chinese expert consensus meeting, China
16. Chinese Ministry of Health, China
17. CRHA, China
18. NHC, China
19. HKUA, China
20. HKSUO, China
21. Instituto Nacional de Cancerología, Colombia
22. Dirección de desarrollo de Servicio de Salud, Costa Rica
23. AMUC, Costa Rica
24. CSMO, Croatia
25. DGCG, Denmark
26. DUCG, Denmark
27. EAU, Europe
28. EANM, Europe
29. ESGO, Europe
30. ESMO, Europe
31. ESO, Europe
32. ESP, Europe
33. ESTRO, Europe
34. ESUR, Europe
35. SIOG, Europe
36. St. Gallen/Vienna, Europe
37. CCAFU, France
38. CNGOF, France
39. AWMF, Germany
40. DKG, Germany
41. DKH, Germany
42. GCFIC, Germany
43. ABSI, India
44. ICMR, India
45. ICON, India
46. Expert group consensus opinion, India
47. APCCC, International
48. ERAS, International
49. ESO, International
50. International expert panel, International
51. ISGyP, International
52. NCCP, Ireland
53. Japanese Urological Association, Japan
54. JSGO, Japan
55. KSGO, Korea
56. KSMO, Korea
57. Lithuanian oncologist, endocrinologist and General practitioners, Lithuania
58. MIMS, Malaysia
59. IMSS, Mexico
60. IKNL, Netherlands
61. NVU, Netherlands
62. Richtlijnendatabase, Netherlands
63. RCGO, Netherlands
64. Ministry of Health from New Zealand, New Zealand
65. Norwegian Gynaecologic Association, Norway
66. IETSI, Peru
67. INEN, Peru
68. PEDEC, Romania
69. SOS, Saudi Arabia
70. SUA, Saudi Arabia
71. SCAN, Singapore
72. CANSA, South Africa
73. Council of Medical Schemes (CMS), South Africa
74. South African Urological Association (SAUA), South Africa
75. SLCOG, Sri Lanka
76. FESEO, Spain
77. SEGO, Spain
78. SEOM, Spain
79. BGCS, UK
80. Joint Guidelines from British Surgical Associations, UK
81. National Health Service, UK
82. The Royal College of Pathologists, UK
83. The Royal College of Radiologists, UK
84. RCOG, UK
85. RCR, UK
86. Scottish Cancer Taskforce, UK
87. American Board of Internal Medicine's, USA
88. American Brachytherapy Society, USA
89. American Society of Plastic Surgeons, USA
90. American Society for Radiation Oncology, USA
91. ACOG, USA
92. ACS, USA
93. ARS, USA
94. ASCO, USA
95. ASTRO, USA
96. AUA, USA
97. SGO, USA
98. SSO, USA
99. IOMPC, Venezuela
