# Supplementary material for: Gender bias in shared decision‐making among cancer care guidelines: A systematic review
Source: Health Expect. 2023 Apr 5;26(3):1019–38. doi: 10.1111/hex.13753 (PMC10154819; doi:10.1111/hex.13753)
Supplement: Supplementary file 3 — Supplementary information. [file HEX-26--s004.docx]

**Appendix 3.**

**The 31-item quality assessment tool used for assessment of shared decision making (SDM) in clinical practice guidelines (CPG) and consensus statements (CS).**

| Domain |  | Item |
| --- | --- | --- |
|  |  |  |
| Basic information | 1 | SDM appears in some section of the CPG |
|  | 2 | SDM appears in the Executive Summary |
|  | 3 | SDM appears in the table of content |
|  | 4 | SDM appears in glossary, abbreviations, acronyms, or topic indexes |
| Background | 5 | SDM basis (concept, benefits, risks, and limitations) are explained |
|  | 6 | Primary affected population is well defined |
|  | 7 | Patients’ subgroups that need special consideration are discussed |
| Evidence selection criteria | 8 | The key (PICO) question related to SDM is specified |
|  | 9 | Details of the strategy used to search for evidence about SDM is reported |
| Evidence strengths & limitations | 10 | Study design(s) and methodology limitations are pondered |
|  | 11 | Appropriateness or relevance of outcomes are considered |
|  | 12 | Consistency of results across studies are detailed |
|  | 13 | Magnitude of benefit versus magnitude of harm is considered |
|  | 14 | Certainty of the supporting evidence on SDM is indicated |
| Recommendations | 15 | Clear and precise recommendations on SDM are provided |
|  | 16 | Distinctive recommendations about SDM for important subgroups are separated |
|  | 17 | Strength of recommendations on SDM is indicated |
| Facilitators and barriers | 18 | Facilitators to SDM application are described |
|  | 19 | Barriers to SDM application are described |
| Implementation advice/tools | 20 | Advice on how recommendations about SDM can be applied in practice is provided |
|  | 21 | Additional materials to support the implementation of SDM are provided |
| Resource implications | 22 | Types of cost of SDM implementation that were considered are specified |
|  | 23 | Information/description of the cost information is provided |
|  | 24 | The information gathered affects recommendations about SDM, and it is well detailed |
| Monitoring/auditing criteria | 25 | Criteria to assess adherence to recommendations about SDM |
|  | 26 | Criteria for assessing impact of implementing these recommendations |
|  | 27 | Advice on the frequency and interval of measurement of these criteria |
| Recommendations & limitations | 28 | Suggestions for further research are provided based on the gaps in the evidence encountered |
|  | 29 | Limitations of the guideline about SDM recommendations are described |
| Editorial Independence & declaration of interest | 30 | Declaration of the value of the SDM use is described |
|  | 31 | Declaration / management of interests (professional, financial, or intellectual) about SDM use is described |
