# Supplementary material for: Gender bias in shared decision‐making among cancer care guidelines: A systematic review
Source: Health Expect. 2023 Apr 5;26(3):1019–38. doi: 10.1111/hex.13753 (PMC10154819; doi:10.1111/hex.13753)
Supplement: Supplementary file 4 — Supplementary information. [file HEX-26--s002.docx]

**Appendix 4**

Identified clinical practice guidelines and consensus statements not published in a journal.

| **Prostate cancer** |
| --- |
| (CMS). Council for Medical Schemes. PMB definition guideline: Prostate cancer. 2020. |
| (SAUA). South African Urological Association. South African prostate cancer guidelines. 2017. |
| (MIMS). Malaysian multi-channel provider of drug information, medical education and services. Prostate Cancer. 2021. |
| (EAU-EANM-ESTRO-ESUR-SIOG). EAU-EANM-ESTRO-ESUR-SIOG. Biochemical recurrence in prostate cancer: The EAU Prostate Cancer Guidelines Panel’s recommendations. 2020. |
| (EAU-ESTRO-ESOR-SIOG). EAU-ESTRO-ESOR-SIOG Guidelines on prostate cancer. 2018. |
| (DUCG). DUCG's National Guidelines for Diagnosis and Treatment of Prostate Cancer, 2015. |
| (AWMF-DKG-DKH). AWMF-DKG-DKH. S3 prostate cancer guideline. 2021. |
| (AWMF). AWMF. PSMA ligand PET/CT in the diagnosis of prostate carcinoma. 2019. |
| (NCCP). NCCP. National Prostate Cancer GP Referral Guideline. 2018. |
| (NCCP). NCCP. Diagnosis, staging and treatment of patients with prostate cancer. National Clinical Guideline No. 8. 2016. |
| (NCCN). Prostate cancer early detection. NCCN Clinical Practice Guidelines in Oncology. 2019. |
| (IKNL). IKNL. Prostate cancer, national guideline version 3.0. 2017. |
| (Zoginstituut Netherlands). Zorginstituut Netherlands. Appropriate use of pharmaceutical products for patients with castration-refractory prostate cancer. 2016. |
| (NVU). NVU. Prostate cancer. 2016. |
| (NICE). The National Institute for Health and Care Excellence. Enzalutamide for treating hormone-sensitive metastatic prostate cancer (technology appraisal guidance TA712). 2021. |
| (NICE). The National Institute for Health and Care Excellence. Darolutamide with androgen deprivation therapy for treating hormone-relapsed non-metastatic prostate cancer (technology appraisal guidance TA660). 2020. |
| (NICE). The National Institute for Health and Care Excellence. Prostate cancer: diagnosis and management. (NICE guideline NG131). 2019. |
| (NICE). The National Institute for Health and Care Excellence. Enzalutamide for hormone-relapsed non-metastatic prostate cancer (Technology appraisal guidance TA580). 2019. |
| (NICE). The National Institute for Health and Care Excellence. Padeliporfin for untreated localised prostate cancer (Technology appraisal guidance TA546). 2018. |
| (NICE). The National Institute for Health and Care Excellence. Memokath-051 stent for ureteric obstruction (Medical technologies guidance MTG35). 2018. |
| (NICE). The National Institute for Health and Care Excellence. Biodegradable spacer insertion to reduce rectal toxicity during radiotherapy for prostate cancer (Interventional procedures guidance IPG590). 2017 |
| (NICE). The National Institute for Health and Care Excellence. Irreversible electroporation for treating prostate cancer. Interventional procedures guidance [IPG572]. 2016. |
| (NICE). The National Institute for Health and Care Excellence. Radium-223 dichloride for treating hormone-relapsed prostate cancer with bone metastases (Technology appraisal guidance TA412. 2016. |
| (NICE). The National Institute for Health and Care Excellence. Cabazitaxel for hormone-relapsed metastatic prostate cancer treated with docetaxel (Technology appraisal guidance TA391). 2016. |
| (NICE). The National Institute for Health and Care Excellence. Degarelix for treating advanced hormone-dependent prostate cancer (Technology appraisal guidance TA404). 2016. |
| (NICE). The National Institute for Health and Care Excellence. Abiraterone for castration-resistant metastatic prostate cancer previously treated with a docetaxel-containing regimen (Technology appraisal guidance TA259). 2016. |
| (NICE). The National Institute for Health and Care Excellence. Abiraterone for treating metastatic hormone-relapsed prostate cancer before chemotherapy is indicated (Technology appraisal guidance TA387). 2016. |
| (NICE). The National Institute for Health and Care Excellence. Enzalutamide for treating metastatic hormone-relapsed prostate cancer before chemotherapy is indicated (Technology appraisal guidance TA377). 2016. |
| (NICE). The National Institute for Health and Care Excellence. Suspected cancer: recognition and referral (NICE guideline NG12). 2015. |
| (CCO). CCO. Multiparametric Magnetic Resonance Imaging in the Diagnosis of Clinically Significant Prostate Cancer. Guideline 27-2 version 2. 2021. |
| (CCA). Cancer Care Alberta. Local prostate cancer. Clinical Practice Guideline GU-012 – Version 3. 2020. |
| (CCA). Cancer Care Alberta. Advanced/Metastatic prostate cancer. Clinical Practice Guideline GU-010 – Version 2. 2020. |
| (BC). British Columbia. Prostate Cancer Part 1: Diagnosis and Referral in Primary Care. 2020. |
| (BC). British Columbia. Prostate Cancer Part 2: Follow-up in Primary Care. 2020. |
| (CCO). Cancer Care Ontario. An Endorsement of the 2018 Guideline on Hypofractionated Radiation Therapy for Localized Prostate Cancer: An ASTRO, ASCO, and AUA Evidence-Based Guideline. 2018. |
| (CCO). Cancer Care Ontario. Guideline for Optimization of Surgical and Pathological Quality Performance for Radical Prostatectomy in Prostate Cancer Management. Evidence-Based Series 17-3 Version 2. 2017. |
| (CCO). Cancer Care Ontario. Cancer Care Ontario Position Statement on Prostate Cancer Screening using the Prostate-Specific Antigen (PSA) Test. 2017. |
| (CCO). Cancer Care Ontario. Bone Health and Bone-Targeted Therapies for Prostate Cancer. Guideline 3-14 Version 2. 2016. |
| (CCA). Cancer Control Alberta. Prostate cancer. 2015. |
| (ACS). ACS. Prostate cancer prevention and early detection. 2019. |
| (AUA). AUA. Castration-resistant prostate cancer Cookson. 2019. |
| (AUA). AUA. Early detection of prostate cancer: AUA guideline. 2018 |
| (AUA-ASTRO-SUO). Clinically Localized Prostate Cancer: AUA-ASTRO-SUO Guideline. 2017. |
| (NCCN). NCCN Clinical Practice Guidelines in Oncology (NCCN Guidelines). Version 3. 2016. |
| (PCFA). PCFA. Clinical practice guidelines: PSA Testing and Early Management of Test-Detected Prostate Cancer. 2016. |
| (MSC). MSC. AUGE Clinical Guidelines. Prostate cancer in patients over 15 years old. 2015. |
| (AMUC). AMUC. Prostate cancer. Risk factors, early detection and PSA: screening, use and correct interpretation. 2018. |
| (IMSS). IMSS. Prostate cancer diagnosis and treatment. Clinical practice guidelines. 2018. |
| (AUNA). AUNA. Clinical practice guideline: prostate cancer. 2019. |
| (IETSI). IETSI. Clinical practice guideline for the screening, diagnosis and treatment of localized and locally advanced prostate cancer. 2021. |
| (INEN). INEN. Clinical Practice Guideline for the early detection, diagnosis, staging, treatment, rehabilitation and follow-up of patients with prostate cancer. 2021. |
| **Endometrial cancer** |
| (CANSA). CANSA. Cancer of the uterus. 2021. |
| (CMS). CMS. PMB definition guideline: Endometrial cancer. 2019. |
| (ICMR). ICMR. Consensus document for management of uterine cancer. 2019. |
| (SLCOG). SLCOG. Management of histologically confirmed endometrial cancer JE/003/21. 2021. |
| (DGCG). Danish Gynecological Cancer Group. Surgical treatment of endometrial cancer. 2021. |
| (DGCG). Danish Gynecological Cancer Group. Guidelines for the referral, diagnosis, treatment, and control of cancer of the uterine corpora. 4th revision of the guideline. 2019. |
| (GB). GB. Cancer early detection policy (KFE-RL). 2020. |
| (GCFIC). GCFIC. Current recommendations for surveillance, risk reduction and therapy in Lynch syndrome patients. 2019. |
| (GGP). GGP. Guideline on the Diagnosis, Treatment, and Follow-up of Patients with Endometrial Cancer. 2018. |
| (AWMF). AWMF. Interdisciplinary Diagnosis, Therapy and Follow-up of Patients with Endometrial Cancer. Guideline (S3-Level, AWMF Registry Nummer 032/034-OL, April 2018) – Part 1 with Recommendations on the Epidemiology, Screening, Diagnosis and Hereditary Factors of Endometrial Cancer. 2018. |
| (AWMF). AWMF. Interdisciplinary Diagnosis, Therapy and Follow-up of Patients with Endometrial Cancer. Guideline (S3-Level, AWMF Registry Number 032/034-OL, April 2018) – Part 2 with Recommendations on the Therapy and Follow-up of Endometrial Cancer, Palliative Care, Psycho-oncological/Psychosocial. 2018. |
| (RCGO-IKNL). RCGO-IKNL. Dutch National Guideline Endometrial Cancer Version 3.1 [Guideline]. 2018. |
| (NGF). NGF. Uterine cancer (endometrial cancer). 2021. |
| (STNF-NCQSG). STNF-NCQSG. Endometrial Cancer Clinical Quality Performance Indicators. 2018. |
| (RCOG-BGCS). RCOG-BGCS. Joint RCOG/BGCS Guidance for Care of Patients with Gynaecological Cancer during the COVID-19 Pandemic. |
| (NHS). NHS. Implementing Lynch syndrome testing and surveillance pathways. 2021. |
| (RCR). RCR. Guidance for radiotherapy for gynaecological cancer and COVID-19. 2020. |
| (NICE). NICE. Testing strategies for Lynch syndrome in people with endometrial cancer. Diagnostics guidance [DG42]. 2020. |
| (BGCS). BGCS. Sentinel Consensus Document for Vulval, Endometrial and Cervical Cancer BGCS. 2019. |
| (RCPATH). RCPATH. Standards and datasets for reporting cancers. Dataset for histological reporting of endometrial cancer. 2017. |
| (GOGG-MCGCNG). GOGG-MCGCNG. Management of uterine cancers. 2016. |
| (PBCN-NHS). PBCN-NHS. Guideline for the Management of Endometrial Cancer Formerly the Guideline for Post Menopausal Bleeding and Endometrial Cancer. 2015. |
| (GCSG-GIG-NHS). GCSG-GIG-NHS. National optimal pathway to endometrial cancer: Point of suspicion to first definitive treatment in adults (aged 16 and over). 2020. |
| (GCSG-GIG-NHS). GCSG-GIG-NHS. All Wales Guideline for the Management of Uterine Cancer. 2019. |
| (CCO). Cancer Care Ontario. Systemic Therapy for Advanced or Recurrent Endometrial Cancer and Advanced or Recurrent Uterine Papillary Serous Carcinoma. 2019. |
| (UHN PMCC). UHN PMCC. Princess Margaret Cancer Centre. Clinical Practice Guidelines. Gynecologic cancer: Endometrial. 2019. |
| (BCCA). BCCA. Endometrium. 2018. |
| (CCO). Cancer Care Ontario. Screening for Lynch Syndrome by Immunohistochemistry BRAF Mutations Analysis and MLH1 Promoter Methylation Analysis for Patients in Ontario with Colorectal or Endometrial Cancers. 2015. |
| (AHS). AHS. Endometrial cancer. Clinical practice guideline GYNE-002 Version 5. 2015. |
| (ASTRO). ASTRO Guideline on the Role of Postoperative Radiation Therapy for Endometrial Cancer. 2017. |
| (ACR). ACR. Adjuvant Management of Early Stage Endometrial Cancer. 2016. |
| (ACOG). ACOG. Practice Bulletin. Clinical management guidelines for obstetrician-gynecologists: Endometrial cancer. 2015. |
| (SGO). SGO clinical practice statement: the role of sentinel lymph node mapping in endometrial cancer. 2015. |
| (CA). CA. Shared follow-up care for women with low-risk endometrial cancer: A guide for General Practitioners (GP Guide). 2020. |
| (CA). CA. Shared follow-up and survivorship care for women with low-risk endometrial cancer: summary of evidence. 2020. |
| (NSWG). NSWG. Gynaecological cancer: A guide to clinical practice in NSW. 2019. |
| (CA). CA. Clinical practice guidelines for the treatment and management of endometrial cancer. 2016. |
| (FASGO). FASGO. Consensus Committee Federación Argentina de Sociedades de Ginecología y Obstetricia F.A.S.G.O. Consenso de Ginecología FASGO 2019 "Endometrial Cancer". 2019. |
| (CIIS). CIIS. Inter-Societies National Consensus on Endometrial Cancer. 2016. |
| (SCGO). SCGO. Endometrial Cancer Management Guideline Protocol  Consensus of the Oncological Gynaecology Branch of the Chilean Society of Obstetrics and Gynaecology. 2018. |
| (ICCR). ICCR. Endometrial cancer histopathology reporting guide. 2017. |
